# Tracking Healthy Days — A Window on the Health of Older Adults

**Published:** 2005-06-15

**Authors:** David G Moriarty, Rosemarie Kobau, Matthew M Zack, Hatice S Zahran

**Affiliations:** Community Health Tracking Unit, Health Care and Aging Studies Branch, Division of Adult and Community Health, National Center for Chronic Disease Prevention and Health Promotion, Centers for Disease Control and Prevention; Health Care and Aging Studies Branch, Division of Adult and Community Health, Centers for Disease Control and Prevention, Atlanta, Ga; Health Care and Aging Studies Branch, Division of Adult and Community Health, Centers for Disease Control and Prevention, Atlanta, Ga; Health Care and Aging Studies Branch, Division of Adult and Community Health, Centers for Disease Control and Prevention, Atlanta, Ga; Dr Zahran was an Association of Teachers of Preventive Medicine fellow when the article was written

## Abstract

In collaboration with its partners in the public health and aging services communities, the Centers for Disease Control and Prevention (CDC) Health Care and Aging Studies Branch has developed and validated a brief set of health-related quality of life (CDC HRQOL) measures for tracking the perceived physical and mental health of adults over time. For the past 12 years, these measures — also called the Healthy Days measures — have been used in an expanding set of population health surveys, surveillance systems, performance report cards, and evaluation studies, and they have provided useful disease and disability burden data to inform decision making and provide new insights for prevention research.

Although now used continuously to assess health-related quality of life for Americans aged 12 years and older, the measures and population data have been especially valuable in applications affecting older adults, for which health-related quality of life is an outcome of primary importance. The CDC HRQOL measures are recommended to public health and social service professionals as a feasible way to assess perceived physical and mental health needs of older adults and to document the effects of policies and interventions.

## Introduction

The physical and mental health status of older adults can range from very healthy to very unhealthy (1). Because improvements in life-saving disease treatments now allow many older adults to live longer with a wide range of chronic health conditions, traditional measures of morbidity and mortality used to monitor the health of older adults are insufficient to characterize the quality of this extended life span. Health-related quality of life (HRQOL) — often characterized as a subjective assessment of physical, mental, and social functioning and well-being — has consequently become an important outcome for public health and clinical medicine.

HRQOL reflects numerous influences from a wide range of individual, social, and environmental factors. Vulnerable populations such as frail elders are especially susceptible to negative influences on their health that are associated with lifelong exposure to risky behaviors (smoking, for example), disability, social isolation, and inadequate housing (2). A key overall goal of both the *Healthy People 2000* and *Healthy People 2010* national health agendas is to improve the HRQOL of the population while increasing overall life expectancy (3). The Centers for Disease Control and Prevention (CDC) and state health agencies have been tracking HRQOL since 1993 to monitor progress toward this goal. What follows is a description of CDC HRQOL surveillance efforts and findings of relevance to program planners and policy makers concerned with the health of older adults.

## Background

[Q]uality of life as an evaluative standard provides a cohesive, transcending concept that can guide the composition, organization, and integration of prevention services for people with chronic disease and for the elderly.

Disability in America, U.S. Institute of Medicine, 1991

In 1988, the CDC established the National Center for Chronic Disease Prevention and Health Promotion to develop public health approaches for addressing the growing burden of chronic illness in the U.S. population. An early priority was to develop and validate a set of HRQOL outcome measures to track the health of adults and assess the effects of public health and social service programs (4). This effort led to assessment tools that could help clarify and resolve a key dilemma of prevention programs targeting older adults — namely, that a longer life is not necessarily a healthier life.

With substantial help from researchers, surveillance experts, and the Association of State and Territorial Chronic Disease Program Directors, CDC staff developed a surveillance definition of HRQOL: "perceived physical and mental health over time." Thus, HRQOL was recognized as a health-oriented subset of the broader concept of overall quality of life, which includes aspects of life satisfaction and happiness (5). HRQOL includes domains of life (e.g., disability, perceived feelings and emotions, social engagement, pain, fatigue) directly influenced by changes in health (6).

### The CDC HRQOL measures

The CDC HRQOL-4 core measures include four questions: one question on self-rated general health status (self-rated health) and three Healthy Days questions about recent physical health, recent mental health, and recent activity limitation. The Healthy Days questions are so named because of their format: for example, the core question on recent physical health is, "Now thinking about your physical health, which includes physical illness and injury, for how many days during the past 30 days was your physical health not good?" The CDC HRQOL-14, a broader set of measures, includes 14 questions: the four CDC HRQOL-4 questions, plus five activity-limitation questions and five additional Healthy Days questions about recent symptoms of pain, depression, anxiety, sleeplessness, and vitality. The activity-limitation questions assess the presence of any self-reported current limitation and, if present, ask about its main cause and duration, as well as whether the help of another person is needed to perform basic activities of daily living or other routine instrumental activities of daily living (7).

### HRQOL surveillance

From 1993 to the present (2005), the CDC HRQOL-4 measures have been included each year in interviews for the state-based Behavioral Risk Factor Surveillance System (BRFSS) (8). These measures were also added to the National Health and Nutrition Examination Survey (NHANES) in 2000, where they are now tracking population HRQOL among adolescents as well as young and older adults at the national level.

The large amount of HRQOL surveillance data acquired through these surveys is also used to identify health-related needs and disparities among older adults, and the data are guiding efforts to improve the HRQOL of older adults. As of January 2005, about 2 million adults had been surveyed in the BRFSS, and close to 25,000 adolescents and adults had been surveyed in the NHANES. As these data are verified and made available in the public domain, they will provide a unique and valuable resource for tracking the perceived health of the community-resident population aged 12 years and older.

## Quality of the CDC HRQOL Measures

[W]e also have to focus more on the quality of life that people live — the prevention of pain and suffering. It's one thing to talk about the number of deaths from pneumonia and influenza, but what about all of the days of illness and hospitalizations that are unnecessary that people experience?

David Satcher, National Press Club, October 7, 1999

In assessing a subjective concept like HRQOL, it is very important to ensure that the measures used are valid — that they measure what they are intended to measure. The measures must also be reliable — they must produce consistent results for an underlying state (e.g., health status) or trait (e.g., optimism). The measures must also be responsive to change — they must be sensitive enough to detect a meaningful change caused by a changed condition or perspective.

The CDC HRQOL measures have been examined for content validity (i.e., the extent to which an instrument reflects the scope of content in question) (4), construct validity (i.e., the extent to which questions are associated with other questions tapping into similar or dissimilar constructs), and criterion validity (i.e., the extent to which an instrument correlates with another accepted criterion) (7).

The CDC HRQOL-14 measures have been shown to be associated, as expected, with demographic characteristics (e.g., age, sex, income levels, education) and disease or disability status (5,8,9).

Several studies have demonstrated acceptable criterion validity for the CDC HRQOL measures in older adult patient and community populations. Examples of such studies include those based on comparisons with the Medical Outcomes Study Short Form-36 among community and disabled adults (8,10), the Health Assessment Questionnaire in a group of rheumatology clinics (11), and subsequent records-based health care use and mortality in a low-income older population (12). Only limited data are available on the responsiveness of these measures (i.e., their ability to detect change over time) in clinical or population studies. However, the strong observed construct validity of the measures and a response format of "how many days in the past 30 days" imply the measures will be responsive when used in intervention studies. In periodic assessments of a nutritional support intervention targeting older Canadians, the measure of physically unhealthy days was found to be responsive to apparent health improvements (13).

Other measurement studies have found the CDC HRQOL measures to be internally consistent (for example, mentally unhealthy days are more strongly associated with sad, blue, or depressed days than with physically unhealthy days) (14) and to have acceptable test–retest reliability (15). Although test–retest reliability was somewhat lower for older than for younger adults, this difference likely resulted from difficulty in question comprehension and recall and also from a real change in perceived health status. In cognitive studies, the questions on measures of "recent days" were more difficult to answer for older adults who had more or highly variable recent health problems and who tried to recall and count the actual number of impaired days (16). However, extensive survey experience with trained interviewers shows that only a small percentage of older adults is unable or unwilling to provide acceptable responses for these measures. For example, from 1993 to 2001, the percentages of adults aged 65 years and older participating in the BRFSS who responded "don't know/not sure" or refused to answer Healthy Days questions were 3.6% for the question on physical health, 2.5% for the question on mental health, and 1.7% for the question on activity limitation.

In addition to having adequate psychometric properties, HRQOL measures must be feasible to use in a variety of settings and formats. Feasibility refers to ease of administration; low cost of administration; and ease of data collection, analysis, and interpretation, and dissemination of results. The CDC HRQOL measures have proved to be easy to use, analyze, and interpret in a variety of public health applications (17). The measures have been used most often in household surveys and health care settings; they have also been used in social service settings in Massachusetts (18) and in long-term care institutions (10). Most CDC HRQOL surveys have been conducted through telephone interviews; the second most common format is in-person interviews. The measures have also been effectively used as self-administered questionnaires (12) and interactive forms via the Internet (19).

Because questionnaire length and respondent burden are important quality and cost factors in survey use, the brevity of the CDC HRQOL-4 and HRQOL-14 offers cost benefits. The questions can be easily added to other surveys and questionnaires, thereby substantially enriching the possibilities for comparisons among populations and across prevention studies.

## Key Surveillance Findings

### HRQOL trends among adults, 1993–2001

People not only want to live a long life, but they also want to enjoy a healthy life. As the baby boom becomes the senior boom, quality of life will become a central issue for our health system. With *Healthy People 2010*, we want to add years to your life and health to your years.

Donna Shalala, HCFA Health Watch, Volume V, No. 5, February 2000

The CDC HRQOL measures reflect the population burden of chronic health conditions, including activity limitations, arthritis, epilepsy, obesity, asthma, depression, stroke, diabetes, and violence (7,8,19-27); perceived unmet health needs and disparities (28-34); and seasonal patterns and time trends (8,34,35). A recent BRFSS-based assessment of HRQOL trends among adults found that overall mean unhealthy days rose from 5.2 days in 1993 to 6.0 days in 2001 (35). This population prevalence increase accounted for an extra 15 million unhealthy years of life during the period of 1994 through 2001 compared with what would have been if the 1993 HRQOL prevalence level had persisted through 2001.

HRQOL also worsened during this period among most socioeconomic and demographic groups — especially among high school graduates without further education and individuals with annual household incomes less than $50,000. In 18 of the 50 states, the mean number of unhealthy days increased; only North Dakota reported a decrease. These trends are not explained by the aging of the adult population during this period because these data were age adjusted.

The average number of overall unhealthy days also increased in each adult age group. The steepest increases were among those aged 45 to 64 years, an important finding that suggests a growing level of unmet health needs in this age group. For older adults, only the mean number of physically unhealthy days increased among those aged 65 to 74 years; mentally unhealthy and activity-limitation days showed no consistent trends. During this period, the percentage of people who reported fair or poor health actually decreased among those aged 65 to 74 years and those aged 75 years and older.

### HRQOL of older middle-aged adults with low household incomes

A 2003 BRFSS study that sought to explain why older middle-aged adults with low annual household incomes reported worse HRQOL than did younger or older adults found that people aged 45 to 64 years with low annual household incomes (<$15,000) reported substantially more physically and mentally unhealthy days than those in the same age group with higher incomes (32). In the low-income group aged 45 to 64 years, employment status and activity limitation accounted for the most variability in overall unhealthy days (34.8% among men and 32.1% among women). Surprisingly, nearly half (48.8%) of the low-income group reported a current activity limitation, and approximately one third (34.6%) of this subgroup reported being unable to work. Although these cross-sectional analyses imply that retirement-age entitlements provide notable HRQOL benefits for adults aged 65 years and older who have low incomes, compared with adults aged 45 to 64 years who have low incomes, substantial HRQOL differences persist among demographic and income subgroups of older adults (31).

### Older adult HRQOL by socioeconomic status and sex

Data from the 2003 BRFSS showed that levels of HRQOL reported by adults aged 65 years and older differed substantially according to socioeconomic status (SES) and sex. Men with high SES (defined as having both a college degree and an annual household income of ≥$50,000) reported 3.5 (95% confidence interval [CI], 2.9–4.1) unhealthy days; women with low SES (defined as having no high school diploma or an annual household income of <$15,000) reported 10.1 (95% CI, 9.6–10.6) unhealthy days (Figure). It is also notable that 2.2 times as many older men as older women were in the high-SES (healthiest) group, whereas women outnumbered men by a factor of 1.9 in the low-SES (least healthy) group (data not shown).

**Figure:**
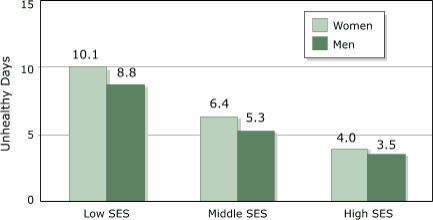
Mean number of unhealthy days among adults aged ≥65 years by level of socioeconomic status (SES) and sex, Behavioral Risk Factor Surveillance System, 2003. Low SES: those without a high school diploma or with an annual household income of <$15,000. High SES: those with a college degree and with an annual household income of ≥$50,000. Middle SES: all other respondents.

**Table:** 

## Other Applications for the CDC HRQOL Measures

In addition to their use in assessing the burden of chronic health conditions, tracking progress on *Healthy People 2010* goals, and identifying health needs and disparities among populations, CDC HRQOL data are now being used for a variety of additional purposes at the national, state, and community levels.

### Comparable measures and data

CDC HRQOL measures have been recommended or adopted for use by various U.S. standards-setting groups, including the Foundation for Accountability — with support from The Robert Wood Johnson Foundation (19). The U.S. Council of State and Territorial Epidemiologists and its partners have also recently adopted each of the CDC HRQOL-4 measures as part of their standard set of 92 chronic disease indicators (36). These recommended indicators are designed to provide public health programs with comparable state-specific surveillance data on key chronic diseases, conditions, and risk factors.

### Summary measures of health

The North Carolina Department of Health and Human Services and the Utah Department of Health have used their CDC HRQOL data to derive the health weights needed for calculating state health expectancy estimates (7,37). To generate these population health estimates, analysts combine age, sex, and other subgroup-specific health weights with mortality rates and demographic data to estimate the remaining years of healthy (and unhealthy) life for the average person in each subgroup.

### Performance report cards

CDC HRQOL surveillance data are also being used in several national and state-based health performance measures, or report cards, including the new CDC and Merck Institute of Aging & Health report, *The State of Aging and Health in America 2004* (38).

### Performance monitoring

The Performance Outcomes Measures Project of the U.S. Administration on Aging uses a crosscutting emotional well-being performance measure that includes Healthy Days questions on recent depression, anxiety, sleeplessness, and vitality (39).

### Health care evaluation

Since 2003, the CDC HRQOL-4 measures have been included in the Medicare Health Outcome Survey, a standardized Health Plan Employer Data and Information Set (HEDIS) measure developed by the National Committee for Quality Assurance for use in evaluating the quality of care delivered by Medicare managed care plans (40).

### Prescription assistance research and evaluation

Pennsylvania's Pharmaceutical Assistance Contract for the Elderly (PACE) program uses the CDC HRQOL-14 measures in mail surveys to assess the quality of its services and identify factors that could improve the health and safety of its clients. The linking of survey data to health care use, prescription use, and mortality records has also provided PACE planners and researchers with findings of substantial benefit to older adults (12,20).

### Other older-adult applications

Two recent volumes of the CDC's *Chronic Diseases Notes and Reports* feature several additional applications of the CDC HRQOL measures, including an article that discusses the relevance of HRQOL surveillance to an aging population (17). The CDC HRQOL Web site also describes national and state HRQOL surveillance and research activities based on the CDC HRQOL measures and provides Internet links to examples of how CDC HRQOL data have been used by states and communities (7).

## Access to Data and Tools

Public-domain copies of the CDC HRQOL-14 measures in English and Spanish are available in the Methods and Measures section of the CDC HRQOL Web site (7).

The CDC HRQOL Web site provides annual BRFSS-based prevalence estimates and 95% CIs for adults' physically unhealthy days, mentally unhealthy days, activity-limitation days, and overall unhealthy days (physically or mentally) for each state and for the nation as a whole from 1993 through 2003. The percentage of adults with fair or poor health and the percentages of adults reporting 14 days or more on the three core Healthy Days measures are also included. If sample sizes are adequate, these prevalence data are further disaggregated by sex, age group, and race/ethnicity. This additional breakdown gives state planners the ability to examine HRQOL patterns within their own states, which might indicate a need to study further and address a potentially unmet health need, disparity, or unfavorable trend.

Electronic copies of anonymous BRFSS survey data — including CDC HRQOL-4 data for all states (and expanded CDC HRQOL-14 data for some states) — are in the public domain. Data files and documentation for each year can be downloaded from the BRFSS Web site, which is linkable from the CDC HRQOL Web site's Resources Page (7). Sample SAS (SAS Institute Inc, Cary, NC), SPSS (SPSS Inc, Chicago, Ill), and SUDAAN (Research Triangle Institute, Research Triangle Park, NC) syntax for computing the Healthy Days summary measure is available in the Methods and Measures section of the CDC HRQOL Web site.

Additional information is available from the HRQOL Tracking Program, Division of Adult and Community Health, National Center for Chronic Disease Prevention and Health Promotion, Centers for Disease Control and Prevention, 4770 Buford Hwy NE, Mail Stop K-51, Atlanta, GA 30341, or by calling 770-488–5464.

## Conclusion

Changes in HRQOL resulting from unavoidable health conditions and life situations are common to all older adults. Accordingly, prevention efforts should focus on developing strategies to minimize decrements in HRQOL (6). Population HRQOL tracking can be used to identify and prioritize population health needs of older adults and address related disparities. It also has the potential to follow improvements over time. The CDC HRQOL measures offer valid, comparable, and useful measures that are feasible to include in health and social surveys and evaluations. These CDC HRQOL measures are recommended to public health and social services professionals to assess perceived physical and mental health needs of older adults and to document the effects of policies and interventions.
